# mSphere of Influence: Ushering in the CRISPR Revolution to *Toxoplasma* Biology

**DOI:** 10.1128/mSphere.00307-19

**Published:** 2019-07-03

**Authors:** Alfredo J. Guerra

**Affiliations:** a Department of Microbiology and Immunology, University of Michigan, Ann Arbor, Michigan, USA

**Keywords:** CRISPR, *Toxoplasma*, genome editing, genome engineering

## Abstract

Alfredo J. Guerra works in the field of molecular parasitology and structural biology. In this mSphere of Influence article, he reflects on how “Efficient Gene Disruption in Diverse Strains of Toxoplasma gondii Using CRISPR/CAS9” by Bang Shen et al. (mBio 5:e01114-14, 2014, https://doi.org/10.1128/mBio.01114-14) and “Efficient Genome Engineering of Toxoplasma gondii using CRISPR/CAS9” by Saima M. Sidik et al. (PLoS One 9:e100450, 2014, https://doi.org/10.1371/journal.pone.0100450) made an impact on him by successfully implementing strategies to genetically manipulate T. gondii using CRISPR/CAS9 gene editing technology.

## COMMENTARY

It is difficult to overstate the extent to which CRISPR/CAS9 (clustered regularly interspaced short palindromic repeats with CAS9) gene editing technology has changed the landscape of genome editing. The field of apicomplexan biology is no exception to this trend. As a relative newcomer to the field of molecular parasitology, there are two papers that strongly influenced my current research in Toxoplasma gondii: “Efficient Gene Disruption in Diverse Strains of Toxoplasma gondii Using CRISPR/CAS9” by Shen et al. (1) and “Efficient Genome Engineering of Toxoplasma gondii Using CRISPR/CAS9” by Sidik et al. (2). Although genetic manipulation of T. gondii has been more manageable than that of related apicomplexan parasites, namely *Plasmodium* spp. and *Cryptosporidium* spp., genetic manipulation in a site-specific manner remained a laborious task and was generally limited to special genetic backgrounds. Both Shen et al. and Sidik et al. probed the idea of adapting CRISPR/CAS9 to genetically manipulate T. gondii. These two papers showed that CRISPR/CAS9 can be used not only to disrupt specific genes by introduction of insertions and deletions via the nonhomologous end-joining (NHEJ) pathway but can also be used to tag specific genes and also generate point mutations in a site-specific manner.

Shen et al. and Sidik et al. spearheaded the implementation of CRISPR/CAS9 for gene inactivation, gene tagging, and insertion of point mutations in T. gondii. Shen et al. adapted a CRISPR/CAS9 system with a single guide RNA (sgRNA) to target genes in T. gondii for efficient gene disruption. Taking advantage of a resistance to fluorodeoxyribose that results upon the deletion of the uracil phophoribosyl transferase (*UPRT*) gene, Shen et al. site-specifically disrupted the *UPRT* locus both by the nonhomologous end-joining (NHEJ) pathway as well as via homologous recombination, where the *UPRT* locus was replaced with a pyrimethamine-resistant dihydrofolate reductase (DHFR). The strength of the paper by Shen et al. lies in showing that this approach can be extended to different strains of T. gondii, thereby expanding the utility of this approach in interrogating the complex biology of this apicomplexan parasite. Similarly, Sidik et al. were able to show the efficient disruption of genes via NHEJ using a CRISPR/CAS9 system with an sgRNA targeting the *SAG1* gene. Sidik et al. then took their system a step further and showed that in a strain that is deficient in NHEJ (Δ*KU80*), it is possible to introduce site-specific point mutations by supplying a repair template harboring the desired mutation. One advantage of this approach is the ability to use relatively short homology arms—typically 40 bp. Sidik et al. leveraged drug resistance that results from the specific point mutations that were introduced to give a simple yet powerful confirmation that the desired mutations were introduced. Finally, Sidik et al. showed that CRISPR/CAS9 can be used to tag endogenous loci with an epitope tag in a Δ*KU80* strain by using a repair template harboring the desired tag flanked by relatively short (∼40-bp) homology arms to target the insertion in frame with the gene of interest.

The tools described in these two papers had a major impact in the T. gondii field as a whole. One impressive follow-up study is a genome-wide CRISPR screen that allowed Lourido and coworkers to determine the degree to which specific genes contribute to the fitness of the parasites in human foreskin fibroblast (HFF) culture (3). The data generated from this screen have been incorporated into ToxoDB and are routinely used as a resource to measure the importance of a particular gene to the overall fitness of the parasite (4). Sibley and coworkers have also followed up their studies and combined CRISPR/CAS9 gene editing with a plant-derived auxin-inducible degron to rapidly downregulate target proteins. My own research was directly influenced by the tools described in these two papers, particularly as part of a structure-function study of the APC_β_ domain of T. gondii perforin-like protein 1 (*Tg*PLP1) (5). In that study, I used a CRISPR/CAS9-based approach to introduce amino acid deletions and point mutations to probe the role of a hydrophobic loop in the *Tg*PLP1 APC_β_ domain in egress from the host cell. Ultimately, CRISPR technology is still relatively new to the field of apicomplexan biology, and there are still enticing applications that are yet to be implemented in these eukaryotic pathogens.
